# Sexual violence against children in the state of Santa Catarina, Brazil: characteristics and factors related to repetitive violence

**DOI:** 10.1590/1984-0462/2023/41/2022069

**Published:** 2023-04-07

**Authors:** Vanessa Borges Platt, Elza Salema Berger Coelho, Carolina Bolsoni, Michele Honicky, Guilherme Platt Bordin, Maria Antônia Vicente de Camargo

**Affiliations:** a Universidade Federal de Santa Catarina, SC, Brazil.; b Universidade do Sul de Santa Catarina, SC, Brazil.

**Keywords:** Sex offenses, Child, Recurrence, Epidemiology, Notification, Violência sexual, Criança, Recorrente, Epidemiologia, Notificação

## Abstract

**Objective:**

The aim of this study was to characterize child sexual abuse and investigate the factors related to its repetition in the state of Santa Catarina, Brazil.

**Methods:**

This is a descriptive and analytical study, with data from 2009-2019 SINAN. Sociodemographic variables related to the circumstances of violence were analyzed. Multivariate logistic regression was used to test factors related to repetitive violence.

**Results:**

A total of 3489 cases of child sexual abuse were reported: 73.3% were girls, the most prevalent age ranged from 6 to 10 years, and 51% reported repetitive violence. The majority was perpetrated by one (85.6%) person, and in cases in which two or more perpetrators were involved, the proportion of occurrence was higher for boys (17%) versus girls (13%). Among the risk factors for the repetition of sexual violence are the place of occurrence being the residence, the perpetrator (the stepfather, the brother, and the father) being under the influence of alcohol, and the age of the child between 6 and 10 years.

**Conclusion:**

The profile and factors that help in the identification of repetitive child sexual abuse were presented, such as the authorship being related to stepfathers, parents, and siblings, the perpetrator being under the influence of alcohol, and the victim’s age between 6 and 10 years.

## RESUMO

**Objetivo:**

Caracterizar a violência sexual contra crianças e investigar os fatores relacionados a sua repetição no Estado de Santa Catarina, Brasil.

**Métodos:**

Estudo descritivo e analítico, com dados do Sistema de Informação de Agravos de Notificação (SINAN) 2009–2019. Foram analisadas variáveis sociodemográficas relacionadas à circunstância da violência. Utilizou-se regressão logística multivariada para testar os fatores relacionados à violência de repetição.

**Resultados:**

Foram notificados 3.489 casos de violência sexual: 73,3% de meninas, com idade mais prevalente de seis a dez anos, e 51% referiram violência de repetição. A maioria dos casos foi perpetrada por um autor (85,6%) e, quando a violência teve dois ou mais envolvidos na autoria, a proporção de ocorrência foi mais elevada para meninos (17%) do que para meninas (13%). Entre os fatores de risco para a repetição da violência estão o local da ocorrência ser a residência, o autor da violência estar alcoolizado, ser ele o padrasto, o irmão e o pai, e a idade da criança estar entre seis e dez anos.

**Conclusões:**

Apresentaram-se o perfil e fatores que auxiliam na identificação da violência sexual por repetição em crianças, como a autoria ser relacionada aos padrastos, pais, irmãos, o autor da agressão estar alcoolizado e a vítima ter entre seis e dez anos.

## INTRODUCTION

Child sexual abuse (CSA) is identified as a violation of human and sexual rights, as it makes it impossible for children to enjoy a sexuality compatible with their stage of development, free from discrimination or coercion.^1^ A specific dynamic is identified in this unhealthy phenomenon of CSA, being insidious at first and becoming more intrusive as the perpetrator gains the trust of their victims. CSA is most often perpetrated by family members, with a decreasing order of incidence as follows: fathers, brothers, mothers, and other caregivers.^2, 3, 4, 5, 6^

CSA causes a series of sequelae including post-traumatic stress disorder, poor school performance, higher risk of psychiatric disorders, and even suicide attempts,^7,8^ which will be worse if this violence is repeated.^7,9^

Although repetitive violence against children is more frequent than an isolated episode,^8^ its real prevalence is unknown, with rates in different surveys ranging around 35% in two international studies.^7,10^ The condition of dependence of minors on their relatives, especially their parents and other residents of the same household, should possibly contribute to the greater occurrence of this type of violence and its underreporting.^4^

However, the relationship between the repetitive CSA and the relationship between the victim and the perpetrator cannot always be measured. One of the reasons may be due to the difficulty in documenting and reporting intrafamilial CSA, as there is a social assumption that the child must be protected by their family. There is also the possibility that victims may be forced to deny accusations of violence perpetrated by relatives out of fear or apprehension about losing contact with their “loved one” or fear of what will happen to them.^3,4^ Thus, knowing the characteristics and risk factors (RF) related to repetitive CSA is important, helping both the professionals who care for these children and the measures that can be taken in the protection and justice systems. This study aimed to characterize the cases of CSA reported in the state of Santa Catarina, in SINAN, and to identify the factors related to its repetition.

## METHOD

This is a cross-sectional study that evaluated all suspected or confirmed cases of CSA, from January 2009 to December 2019, reported in the state of Santa Catarina (SC), in SINAN. SC is the 20th largest state in Brazil, with an estimated population of 7,164,788 inhabitants in 2019, with a population of 842,530 children (under 10 years old).^11^

The exposure variables related to the victim were age (categorized as 0–2 years, 2–6 years, and 6–10 years), gender (female or male), the presence/absence of disabilities, and skin color followed the self-reference of victim/informant according to the options used in the 2010 Census of the Brazilian Institute of Geography and Statistics, namely, white, black, yellow, brown, or indigenous.^11^ The place of occurrence of sexual violence (SV) was categorized into residence (residence and collective housing) and other places.

The characteristics of the likely perpetrator of the SV were gender (male and female), number of people involved (1 or ≥2 involved), suspected alcohol use (if yes or no), and life cycle (child, 0–9 years old; adolescent, 10–19 years; young, 20–24 years; adult, 25–59 years; elderly, 60 years or more). For further logistic regression analysis, because age “25–59 years” is at risk, all other ages were grouped, generating the category “all other ages.” It was also categorized in relation to the bond or degree of kinship with the victim: father, mother, stepfather, stepmother, boyfriend, ex-boyfriend, brother, friend, caregiver, stranger, person in an institutional relationship, or others (uncle, cousin, stepfather, the grandmother’s new partner; grandfather, grandmother, and stepchild of the father/mother). The variables of the link that presented n<50 were grouped. To facilitate the interpretation, the category “known” was also generated, that is, “being known to the victim”, and it was composed of the variables listed above.

SV was typified in terms of the presence or absence of sexual harassment, sexual exploitation, pornography, indecent assault, and rape.

The outcome variable, “repetitive sexual violence”, was determined by the occurrence of more than one episode of SV (“two or more times”) and was categorized as “once”, translating an isolated case. For the present study, we chose to use the definitions of repetition and recurrence as synonyms.

The data obtained were extracted from SINAN electronic spreadsheets in Microsoft Excel^®^ format and evaluated by two observers. To characterize the CSA, data were analyzed using descriptive statistics in relative and absolute frequencies and 95% confidence intervals (CIs). To compare the characterization of victims and perpetrators and their specifications according to gender, chi-square test and Fisher’s exact test were performed. To analyze the factors related to repetitive SV, a bivariate analysis was performed using the bivariate logistic regression test.

The specified relationship between the child and the author of the SV was also investigated by means of multivariate logistic regression, with input of variables using the backward procedure. Variables that presented p<0.20 in the bivariate analysis were included in the adjusted analysis and those that presented multicollinearity were excluded from the model. The analyses were adjusted for number involved and disability (set 1) and number involved, disability, gender, and race (set 2). In the logistic regression analyses, the results were expressed as an odds ratio (OR) and the respective 95%CI.

Statistical analyses were performed using the Statistical Package for the Social Sciences, version 23.0. For all analyses, p<0.05 was considered significant.

The study was approved by the Research Ethics Committee of the institution of origin of the researchers, under Embodied Opinion n. 3,615,628/2019.

## RESULTS

In SINAN, 3489 cases of CSA were reported. The database used was previously evaluated by the author and had no duplication, with adequate data consistency and completeness of information.

Most abused children were female (73.3%), white (83.4%), did not have a disability (96.9%), and suffered violence at home (77.8%). Age (p<0.008) and the presence of disability (p<0.006) show a significant difference according to the victim’s gender (p<0.05). In the typification of violence, it was observed that rape was almost three times more frequent in females, compared to males, but when SV was more invasive, accompanied by penetration, the highest prevalence occurred with males.

Sexual harassment (p<0.001) and the existence of penetration — anal (p<0.001) and oral (p=0.01) — showed a significant difference according to the victim’s gender (Table 1).

**Table 1.** Characterization of victims and typology of child sexual abuse and gender, Santa Catarina, Brazil, 2009–2019 (n=3,489).

	Total	Female (n=2,557)		Male (n=932)		p-value*
	n (%)	n (%)	95%CI	n (%)	95%CI	
Age (years)						
<2	317 (9.1)	253 (9.9)	8.8–11.1	64 (6.9)	5.4–8.7	**0.008**
2–6	1,738 (49.8)	1,280 (50.1)	48.1–52.0	458 (49.1)	45.9–52.4	
6–10	1,434 (41.1)	1,024 (40.0)	38.2–42.0	410 (44.0)	40.8–47.2	
Race^†^ (n=3,372)						
White	2,825 (83.8)	2,064 (83.4)	81.8–84.8	761 (84.9)	82.4–87.1	0.561^‡^
Mixed	353 (10.4)	263 (10.6)	9.5–11.9	90 (10.0)	8.2–12.2	
Black	146 (4.3)	111 (4.5)	3.7–5.4	35 (3.9)	2.8–5.4	
Indigenous	36 (1.1)	30 (1.2)	0.8–1.7	6 (0.7)	0.3–1.5	
Yellow	12 (0.4)	8 (0.3)	0.2–0.6	4 (0.5)	0.2–1.2	
Deficiency^†^ (n=3,341)						
No	3,219 (96.3)	2,369 (96.9)	96.1–97.5	850 (94.9)	93.2–96.1	**0.006**
Yes	122 (3.7)	76 (3.1)	2.5–3.9	46 (5.1)	3.9–6.8	
Number of times^†^ (n=2,620)						
Once	1,287 (49.1)	936 (49.2)	46.9–51.4	351 (49.0)	45.4–52.7	0.950
2 or more	1,333 (50.9)	968 (50.8)	48.6–53.1	365 (51.0)	47.3–54.6	
Sexual harassment^†^ (n=3,236)						
Yes	1,463 (45.2)	1,117 (47.3)	45.3–49.3	346 (39.6)	36.4–42.9	**<0.001**
No	1,773 (54.8)	1,245 (52.7)	50.7–54.7	528 (60.4)	57.1–63.6	
Pornography^†^ (n=3,182)						
Yes	125 (3.9)	84 (3.6)	2.9–4.5	41 (4.7)	3.5–6.4	0.150
No	3,057 (96.1)	2,233 (96.4)	95.5–97.0	824 (95.3)	93.6–96.5	
Sexual exploitation^†^ (n=3,224)						
Yes	121 (3.8)	96 (4.1)	3.4–5.0	25 (2.8)	1.9–4.2	0.098
No	3,103 (96.2)	2,250 (95.9)	95.0–96.6	853 (97.2)	95.8–98.1	
Rape^†,§^ (n=3,212)						
Yes	2,459 (73.9)	1,717 (73.5)	71.7–75.3	656 (74.9)	71.9–77.7	0.633
No	839 (26.1)	619 (26.5)	24.7–28.3	220 (25.1)	22.3–28.1	
Others^†,//^ (n=3,245)						
Yes	1,578 (48.6)	1,199 (49.4)	48.6–52.6	379 (43.2)	39.9–46.4	**<0.001**
No	1,667 (51.4)	1,168 (50.7)	51.3–51.4	499 (56.8)	53.5–60.0	
Penetration^†^ (n=928)						
Yes	526 (56.7)	303 (48.6)	44.7–52.6	223 (73.1)	67.8–77.8	**<0.001**
No	402 (43.3)	320 (51.4)	47.4–55.3	82 (26.9)	22.2–32.2	
Type of penetration^†^						
Anal (n=1,031)						
Yes	305 (29.6)	75 (10.7)	8.6–12.3	230 (69.3)	64.1–74.0	**<0.001**
No	736 (70.4)	624 (89.3)	86.7–91.4	102 (30.7)	26.0–35.9	
Oral (n=1,013)						
Yes	107 (10.6)	61 (8.8)	6.9–11.2	46 (14.3)	10.9–18.6	**0.008**
No	906 (89.4)	631 (91.2)	88.8–93.1	275 (85.7)	81.2–89.1	
Vaginal (n=682)						
Yes	281 (24.3)	281 (41.2)	37.6–44.9	NA	–	NA
No	878 (75.7)	401 (58.8)	55.1–62.2			

95%CI: 95% confidence interval; *Chi-square test; ^†^Data without information from all records; ^‡^Fisher’s exact test; ^§^Association of violent assault on indecency and rape; ^//^Association of sexual harassment, pornography, and sexual exploitation. Bold indicates statistically significant p-values.

Most of the perpetrators were men (92.0%) and of these, the victims’ acquaintances predominated (94.1%). Being under the influence of alcohol (p=0.004) and the life cycle of the perpetrator (p<0.001) showed a significant difference according to the victim’s gender (p<0.05). However, the majority (83.0%) of the sexual offenders were not under the effect of alcohol when they sexually victimized the children (Table 2). The main bonds of the perpetrators were parents (21.8%), stepfathers (12.2%), and uncles (8.6%). Female victims were more frequently abused than male for all the authorship bonds described. This association between perpetrators and the victim’s gender was statistically significant (p<0.05) when the bond with the victim was father, stepfather, cousin, and grandmother (Table 3).

**Table 2.** Characterization of the perpetrators of child sexual abuse, according to the gender of the victims Santa Catarina, 2009–2019 (n=3,215).

	Total	Female (n=2,557)		Male (n=932)		p-value*
	n (%)	n (%)	95%CI	n (%)	95%CI	
Gender^†^						
Male	2,956 (84.7)	2,150 (92.0)	90.8–93.0	806 (91.8)	89.8–93.4	0.408
Female	134 (3.8)	92 (3.9)	3.2–4.8	42 (4.8)	3.6–6.4	
Both	125 (3.9)	95 (4.1)	3.3–4.9	30 (3.4)	2.4–4.8	
Acquaintance of the victim^‡^ (n=3,064)						
Yes	2,880 (94.1)	2,085 (93.5)	92.4–94.5	795 (95.2)	93.5–96.5	0.083
No	184 (5.9)	144 (6.5)	5.5–7.6	40 (4.8)	3.5–6.5	
Alcohol use^†^ (n=2,378)						
Yes	378 (15.9)	294 (17.2)	15.5–19.1	84 (12.5)	10.2–15.2	**0.004**
No	2,000 (84.1)	1,412 (82.8)	80.9–84.5	588 (87.5)	84.8–89.8	
Life cycle^†,§^ (n=1,854)						
Other ages	688 (37.1)	448 (32.3)	29.9–34.8	240 (51.5)	46.9–56.0	**<0.001**
20–59 years old	1,166 (62.9)	940 (67.7)	65.2–70.1	226 (48.5)	44.0–53.1	
Number of involved^†^ (n=3,158)						
1	2,702 (85.6)	1,981 (86.5)	85.1–87.9	721 (83.0)	80.3–85.3	**0.011**
2 or more	456 (14.4)	308 (13.5)	12.1–14.9	148 (17.0)	14.7–19.7	

95%CI: 95% confidence interval; *Chi-square test; ^†^Data without information from all records; ^‡^Combination of the categories father, mother, stepfather, stepmother, boyfriend, ex-boyfriend, brother, friends, caregiver, friend, person in institutional relationship, and others (uncle, cousin, grandfather, grandfather, grandmother, grandmother’s companion or stepchild of the father/mother); ^§^Junction of age groups: age from 0 to 24 years and 60 years or more;. Bold indicates statistically significant p-values.

**Table 3.** Relationship between perpetrator and victim of child sexual abuse according to the gender of the victims; Santa Catarina, 2009–2019 (n=3,215).

	Total	Female (n=2,557)		Male (n=932)		p-value*
	n (%)	n (%)	95%CI	n (%)	95%CI	
Father^†^ (n=3244)						
Yes	706 (21.8)	554 (23.5)	21.8–25.2	152 (17.2)	14.9–19.9	**<0.001**
No	2,536 (78.2)	1,807 (76.5)	74.8–78.2	731 (82.8)	80.1–85.1	
Mother^†^ (n=3264)						
Yes	120 (3.7)	93 (3.9)	3.2–4.8	27 (3.0)	2.1–9.8	0.241
No	3,144 (96.3)	2,284 (96.1)	95.2–96.8	860 (97.0)	95.6–97.9	
Stepfather^†^ (n=3276)						
Yes	400 (12.2)	315 (13.2)	11.9–14.6	85 (9.6)	7.8–11.7	**0.005**
No	2,876 (88.8)	2,072 (86.8)	85.4–88.1	804 (90.4)	88.3–92.2	
Stepmother^†^ (n=3287)						
Yes	15 (0.5)	13 (0.5)	0.3–0.9	2 (0.2)	0.1–0.8	0.227
No	3,272 (99.5)	2,381 (99.5)	99.1–99.7	891 (99.8)	99.1–99.9	
Brother^†^ (n=3272)						
Yes	156 (4.8)	103 (4.3)	3.6–5.2	53 (6.0)	4.6–7.7	0.050
No	3,116 (95.2)	2,280 (95.7)	94.8–96.4	836 (94.0)	92.3–95.4	
Unknown^†^ (n=3251)						
Yes	210 (6.5)	165 (6.9)	6.0–8.1	45 (5.1)	3.8–6.7	0.051
No	3,041 (93.5)	2,201 (93.1)	91.9–94.0	840 (94.9)	96.2–93.3	
Uncle^†^ (n=3489)						
Yes	300 (8.6)	225 (8.8)	7.8–10.0	75 (8.1)	6.5–10.0	0.483
No	3,189 (91.4)	2,332 (91.2)	90.0–92.2	857 (91.9)	90.0–93.5	
Cousin^†^ (n=3489)						
Yes	146 (4.2)	85 (3.3)	2.7–4.1	61 (6.5)	5.1–8.3	**<0.001**
No	3,343 (95.8)	2,472 (96.7)	95.9–97.3	871 (93.5)	91.7–94.9	
Grandmother^†^ (n=3489)						
Yes	114 (3.3)	97 (3.8)	3.1–4.6	17 (1.8)	1.1–2.9	**0.004**
No	3,375 (96.7)	2,460 (96.2)	95.4–96.9	915 (98.2)	97.1–98.9	
Grandfather^†^ (n=3489)						
Yes	67 (1.9)	51 (2.0)	1.5–2.6	16 (1.7)	1.1–2.8	0.597
No	3,422 (98.1)	2,506 (98.0)	97.4–98.5	916 (98.3)	97.2–98.9	
Grandmother companion (n=3489)						
Yes	66 (1.9)	48 (1.9)	1.4–2.5	18 (1.9)	1.2–3.0	0.917
No	3,423 (98.1)	2,509 (98.1)	97.5–98.6	914 (98.1)	97.0–98.8	
Caregiver (n=3257)						
Yes	112 (3.4)	78 (3.3)	2.6–4.1	34 (3.8)	2.8–5.3	0.445
No	3,145 (96.6)	2,293 (96.7)	95.9–97.4	852 (96.2)	94.7–97.2	
Institutional relationship^†^ (n=3282)						
Yes	30 (0.9)	25 (1.0)	0.7–1.5	5 (0.6)	0.2–1.3	0.197
No	3,282 (99.1)	2,368 (99.0)	98.5–99.3	884 (99.4)	98.7–99.8	
Neighbor^†^ (n=3489)						
Yes	41 (1.2%)	27 (1.1)	0.7–1.5	14 (1.5)	0.9–2.5	0.279
No	3,448 (98.8)	2,530 (98.9)	98.5–99.3	918 (98.5)	97.5–99.1	

95%CI: 95% confidence interval; *Chi-square test; ^†^Data without information from all records. Bold indicates statistically significant p-values.

Repetitive CSA was verified in 1,333 cases, representing 75.1% of the total notifications. In the multivariate analysis, after adjusting (Table 4), repetition SV is 3.3 times more likely to occur when the perpetrator is linked to the stepfather, 2 times more when the sibling is involved, and 1.7 times when this was the father. The chance decreases by 0.3 times if the perpetrator is a stranger. The place of violence being the residence, the victim between 6 and 10 years old, and the perpetrator being under the influence of alcohol were also associated with repetitive violence, with a risk of 1.4, 2, and 1.7 times, respectively.

**Table 4.** Factors associated with repeated sexual violence in cases of child sexual abuse reported in SINAN, Santa Catarina, 2009–2019.

	Not adjusted		Adjustment 1		Adjustment 2	
	OR (95%CI)	p-value	OR (95%CI)	p-value	OR (95%CI)	p-value
Author father						
No	1		1		1	
Yes	1.44 (1.19–1.75)	**<0.001**	1.68 (1.29–2.17)	**<0.001**	1.68 (1.29–2.19)	**<0.001**
Author stepfather						
No	1		1		1	
Yes	2.97 (2.27–3.88)	**<0.001**	3.27 (2.35–4.55)	**<0.001**	3.26 (2.34–4.55)	**<0.001**
Author unknown						
No	1		1		1	
Yes	0.21 (0.14–0.30)	**<0.001**	0.30 (0.18–0.51)	**<0.001**	0.30 (0.17–0.52)	**<0.001**
Author brother						
No	1		1		1	
Yes	1.96 (1.33–2.89)	**0.001**	2.00 (1.30–3.07)	**0.002**	1.95 (1.26–3.00)	**0.003**
Author uncle						
No	1		1		1	
Yes	1.28 (0.98–1.69)	0.074	1.39 (0.99–1.96)	0.059	1.41 (0.99–2.00)	0.056
Grandmother’s companion						
No	1		1		1	
Yes	1.66 (0.95–2.90)	0.076	2.06 (1.00–4.23)	0.049	2.04 (0.99–4.20)	0.053
Author cousin						
No	1		1		1	
Yes	1.32 (0.91–1.92)	0.139	1.41 (0.89–2.24)	0.148	1.47 (0.91–2.35)	0.112
Local						
Any place	1		1		1	
Residence	2.05 (1.69–2.49)	**<0.001**	1.39 (1.08–1.79)	**0.011**	1.44 (1.11–1.87)	**0.006**
Victim’s age (years old)						
<6	1		1		1	
6–10	2.03 (1.74–2.38)	**<0.001**	2.02 (1.66–2.45)	**<0.001**	2.02 (1.66–2.47)	**<0.001**
Author – alcohol use						
No	1		1		1	
Yes	2.02 (1.58–2.59)	**<0.001**	1.69 (1.28–2.23)	**<0.001**	1.65 (1.24–2.19)	**0.001**

SINAN: Notifiable Diseases Information System; OR: odds ratio; 95%CI: 95% confidence interval; Adjustment 1: number of people involved (1 to >1) and disability (yes and no); Adjustment 2: number of people involved (1 to >1) and disability (yes and no), sex (male and female), and race (white, black, yellow, mixed race, and indigenous). Homer and Lemeshow: Adjustment 1: 0.960 (1×=58.1; + of 1×=66.4; overall=62.3); Adjustment 2: 0.825 (1×=58.0; + of 1×=67.0; overall=62.6). Bold indicates statistically significant p-values.

When the model was adjusted by the number of people involved (one or more than one) and the presence or absence of disability by the victim (adjustment 1), the Hosmer & Lemeshow test, which measures the accuracy of the model, was 0.96, explaining 58.3% of positive data (Table 4).

## DISCUSSION

In Brazil, 58,030 children were victims of SV in the period from 2011 to 2017, with a predominance of female victims (74.2%), according to the Epidemiological Bulletin of the Ministry of Health (MS-BR).^12^ In 2018, 32,000 cases of CSA were recorded, the highest number since 2011, indicating that more than three cases were recorded per hour. Among these cases recorded by the MS-BR, two-thirds occurred indoors. In one out of every four cases, the perpetrators were part of the victim’s circle of friends or acquaintances, and in 23% of these, the father or stepfather was the perpetrator of the aggression.^13^

Regarding the general characteristics of the children reported as victims of CSA in the present study, females were almost three times more affected than males, in line with what was pointed out in the international global prevalence of CSA, of 15.0–20.0% for girls and 5.0–10.0% for boys.^14^ The most frequent age group were preschoolers, which differs from the literature that points out the school age group as the most frequently victimized,^4–6^ followed by the preschooler.^6^

The most affected age group identified in this sample leads us to reflect that younger children can be easier targets for perpetrators, either because of their physical and emotional fragility or because of the lack of knowledge of the nature of violence, all of this allowing the repetition of violence and impunity for the attacker.

Still, in the characterization of the victims, it was observed that most were white (Caucasian) and did not have a disability. In Brazil, having brown/black skin color is listed as a RF for CSA, a fact not observed in a study in Southern Brazil, and justified by the predominance of the white population in the region,^15^ in agreement with the present study.

The presence of intellectual disability is identified as an RF for SV^2,16^ with a relative risk of 4–8 times greater in children in relation to nondisabled children in a study in Denmark.^16^ This fact is not observed in the present study. This could possibly be justified by the difficulty for victims with disabilities to communicate the fact and the lack of knowledge of the SV. The greater identification of SV in children with disabilities in Denmark may be due to the investment both in specialized child care services and in the support for their parents, which can reduce their burden and help them identify abuse.

It is a consensus in the literature that most perpetrators of CSA are male,^15,17–19^ as observed in this study (84.7%), but women are also involved in CSA (3.8%), but its real prevalence is unknown due to underreporting involving female perpetrators.

Regarding the life cycle of the perpetrator, most were adults, that is, between 24 and 59 years old. However, those who abused male victims had a different age from this age group, with statistical significance. Two studies evaluated the age of abusers: one showed a mean age of 23.3 years,^20^ and the other that addressed the repetitive SV found that the age of abusers was 46 years.^7^

The perpetrator initially provides special attention to the child, thereby earning their trust. After a period, the relationship begins to become sexualized, violating the child’s privacy, initiating conversations and contact with sexual connotation. The perpetrator tries to justify their behavior with the excuse that these experiences are normal.^21^ As a result, SV episodes become more repetitive and intrusive.

Unlike what happens when the victim is an adult, in the case of children, the perpetrator is known to the victims and has a bond with them 85–95% of the time.^15,17,18,21^ The order of frequency in this study was father, stepfather, uncle, brother, and cousin, corroborating the literature.^2,20^

The family is recognized as an important social network of reference for human development, considered a primordial element for the protection of its components. However, in CSA, a paradox is observed: the perpetrator is often a family member who should help protect this child and not violate them.

At the same time, the lack of family life (being an orphan, living in a non-nuclear family environment, or living in a single-parent family) is pointed out by the literature as an RF for CSA.^21,22^ It is observed that it is extremely important to know the family profile of victims of violence to help better understand this prevalent public health problem and provide better care services.^17^

It is not by chance that the victim’s or perpetrator’s house is cited by several researchers as the predominant place of occurrence of CSA.^4,15^

As for the number of perpetrators, in this study, it was related to a single person 85.6% of the time who was not under the influence of alcohol (84.1%), regardless of the victim’s gender, which is in line with other national studies.^3,15^

In this study, rape was the most prevalent type of abuse (70.5%), with penetration being more frequent in males, as in the study by Platt et al.,^15^ but differing from other authors who list SV with penetration being more frequent in females.^2,23^ In a study in Brazil on CSA, those crimes were classified as libidinous acts (7.0%), anal penetration (3.2%), vaginal penetration (1.6%), and others (0.3%).^4^

The information “if it occurred at other times,” in field 53 of the notification form, was essential for assessing the repetitive CSA. This information was filled in 75.1% of the time during the study period. A percentage similar to that was found by Sinanan in a study carried out in seven states of the USA^10^ and higher than that by Machado et al. in a study carried out in Brazil.^24^ For not filling in this information in its entirety, it is necessary to consider the possibility that the memory bias of the age group studied and the fact that disclosure and notification are separate events and often occur years after the violence, making it difficult to obtain information regarding the number of times it happened.^25^ Another factor is that these data are provided by an adult responsible for the child to the professional who notifies. This adult may be unaware of the fact or want to omit the precise information, either out of shame for what happened or because they are protecting the perpetrator.

In the present study, the prevalence of recurrence was 50.9%. There is no consensus in the literature on these data, perhaps because of the factors presented above or even the methodological differences between the studies or the small number of studies that address this issue.^7,10,26^ In Taiwan, Hu et al., whose recurrence was also defined by more than one episode experienced by the same child, found a 35.2% prevalence of CSA after analyzing 91 cases.^7^ While another study in the USA evaluated a new episode in the same child, after 5 years the recurrence was 3.6%,^14^ and the third study did not address the prevalence.^10^ It is, therefore, necessary that more studies be made to measure this sad problem that affects all societies in the world, not sparing gender, age, or social class, and which has an underreporting rate of around 30%.^14^

When analyzing the characteristics of victims of repetitive CSA, no association was found with the victim’s gender; therefore, girls and boys are equally at risk of suffering repetitive CSA. However, characteristics such as being in the age group of 6–10 years were associated with a higher risk of a repetitive CSA, as observed by Sinanan^10^, Palusci and Ilardi.^26^ When the victim is younger, the abuse is more intrusive and the maintenance of secrecy takes place longer, favoring the perpetrator’s impunity.^21^

The responsibility for the occurrence being attributed to the stepfather, father, and brother was associated with a higher risk of repetitive CSA. For Hu et al., this recurrence was also associated with the fact that the perpetration was committed by a family member, with a 4.5 times greater risk when compared to a nonfamily member.^7^ Palusci and Ilardi also studied the repetitive CSA and observed that, in a quarter of them, the same perpetrator was mentioned, usually the parents,^26^ people who should promote and maintain care environments so children reach their full developmental potential without being harmed by any form of abuse or neglect.^18^ In an attempt to explain the factors that contribute to the risk of parents perpetrating CSA to their children, the following are cited: substance abuse, a parental history of CSA or poor parental bonding, and psychiatric problems.^18^

In none of the studies found addressing the recurrence of this condition was the information on the use of alcohol by the perpetrator analyzed. In the present study, it is observed that the risk of a child having been a victim of repetitive CSA is 1.7 times greater if the perpetrator is under the influence of alcohol, when compared to children abused by people who were not under this condition. It is recognized that the use of licit and illicit substances is a RF for committing SV,^18,19^ but the reliability of this information must be considered when the victims are young and do not identify or even verbalize the use of alcohol by their perpetrator.

The risk is 1.4 times greater for repetitive CSA to occur when the location of the violence is the victim’s or perpetrator’s home. These data are lower than those found by Hu et al., whose OR was 3.41.^7^ Even so, the finding is revolting, as it reveals the loss of the protective environment of the residence and signals the presence of the traditional “private barrier” between the domestic and public spheres with regard to the impediment to the disclosure and notification of cases of VS.

Repetitive CSA has a negative impact on children’s lives, with loss of quality of life due to pain and suffering, greater use of the health system,^18^ early onset of sexual activity, greater chance of having risky sexual behavior, such as increased number of partners, and addiction to illicit drugs.^27^ Furthermore, there is low self-esteem,^27^ decreased school performance,^10^ and a high chance of developing psychiatric disorders, as a protection mechanism against disturbing and contradictory emotions caused by recurrent sexual trauma.^28^ As this violence in children is not usually associated with visible physical alterations, the clinical-epidemiological characteristics verified in the present study that can contribute to the detection of repetitive CSA are the perpetrator of the abuse being a family member (the father, stepfather, or brother) and being under the influence of alcohol; the violence occurring in the victim’s or perpetrator’s residence; and if the age of the victim is between 6 and 10 years. If the aforementioned characteristics are observed, they must alert the professionals on the possibility that the child is suffering from repetitive CSA.

In conclusion, approximately half of the 3489 CSA cases notified in SINAN over a period of 10 years had repetitive violence, identifying the following factors: children aged between 6 and 10 years, occurrence in the residence, the perpetrator being known to the victim, especially the stepfather, father, or brother, and the perpetrator being under the influence of alcohol.

The use of secondary data from an official national database is recognized as a limitation to this study. We tried to resolve this limitation with the prior validation of the records,^29^ checking the fields of the variables studied, one by one, by two observers.

It is urgent that different sectors of society, constituents of the system of guaranteeing the rights of children, turn their attention to this sad scourge of society and seek effective actions to curb CSA, protecting them from the possibility of repetition.
